# Clinical Characteristics and Prognosis of Patients with Vasospastic Angina Subjected to the Spasm Provocation Test and the Unavoidable Use of Nitroglycerin

**DOI:** 10.3390/jcdd10010016

**Published:** 2023-01-02

**Authors:** Hiroki Teragawa, Chikage Oshita, Yuko Uchimura

**Affiliations:** Department of Cardiovascular Medicine, JR Hiroshima Hospital, 3 Chome-1-36, Futabanosato, Higashi-ku, Hiroshima 732-0057, Japan

**Keywords:** coronary spasm, coronary spastic angina, multi-vessel spasm, unavoidable use of nitroglycerin, vasospastic angina

## Abstract

Background: Multi-vessel spasm (MVS) has a prognostic impact in patients with vasospastic angina (VSA). Thus, the presence of coronary spasm in both the left coronary artery (LCA) and right coronary artery (RCA) should be assessed through the spasm provocation test (SPT). Nitroglycerin (NTG) is used to avoid SPT-related complications; however, this unavoidable use of NTG may decrease the detection of MVS. Therefore, we investigated the frequency of the unavoidable use of NTG during SPT and clarified the clinical characteristics in patients with VSA who underwent the unavoidable use of NTG during STP. Methods: A total of 141 patients with positive SPT were evaluated. A positive SPT was defined as > 90% constriction in epicardial coronary arteries in response to acetylcholine, accompanied by the usual chest symptoms and/or ischaemic ST-T changes on electrocardiography. When a coronary spasm occurred, we usually wait for the spontaneous relief of the coronary spasm. However, if a prolonged coronary spasm or unstable haemodynamics occurred, 0.3 mg NTG was administered intracoronarily to promptly relieve the coronary spasm and this was defined as the unavoidable use of NTG. Even when the unavoidable use of NTG was administered in one coronary artery, an additional SPT was performed on another coronary artery. If a coronary spasm occurred in another coronary artery, a positive SPT was diagnosed. In contrast, if a coronary spasm was not induced after the unavoidable use of NTG, the judgement was classified as undiagnosed. The patients were divided into two groups according to the unavoidable use of NTG: U-NTG (n = 42) and the final use of NTG: F-NTG (n = 99). The clinical characteristics and frequencies of MVS (≥2 major coronary arteries in which a coronary spasm was provoked) and complications (malignant arrhythmia and unstable haemodynamics requiring catecholamines) during the SPT were compared between the groups. Results: Except for smoking status, all other clinical characteristics did not differ significantly between the groups. More current smokers were observed in the U-NTG group (29%) than in the F-NTG group (12%, *p* = 0.02). The frequency of MVS did not vary significantly between the groups (*p* = 0.28), with 64% for U-NTG and 55% for F-NTG. No significant difference was found between the groups in the frequency of severe complications during SPT (*p* = 0.83), with 2% for U-NTG and 3% for F-NTG. In the U-NTG group, the positive induction rate of coronary spasm in another coronary artery was 40% (17/42). **Conclusions:** The unavoidable use of NTG occurred in ~30% of patients with VSA, most of whom were current smokers. It did not decrease the detection of MVS and potentially prevented severe complications during SPT. Therefore, the unavoidable use of NTG is acceptable during SPT. However, an additional test may need to be performed to assess the presence of MVS.

## 1. Introduction

Vasospastic angina (VSA) is characterised by the transient vasoconstriction of the epicardial coronary arteries, leading to myocardial ischaemia [1,2]. The overall prognosis of patients with VSA was relatively good under medication with coronary vasodilators [3]. Nonetheless, several risk factors for VSA have been reported, including smoking, taking beta-blockers, presence of variant angina (VAP) or angiographic findings including atherosclerosis, focal spasm and multi-vessel spasm (MVS) [3,4,5,6,7,8]. Among such prognostic risk factors responsible for VSA, MVS is particularly important in patients who have undergone the spasm provocation test (SPT). In the SPT, to confirm the presence of MVS, spasm provocation is ideally performed in both the left (LCA) and right (RCA) coronary arteries, with an intracoronary injection of nitroglycerin (NTG) administered at the end of the test. However, in the clinical setting, we have experienced many cases in which a prolonged coronary spasm and haemodynamic instability necessitate the use of NTG to relieve the spasm, even during SPT.

Considerable interest has been recently focused on SPT after an intracoronary infusion of NTG [9,10]. In Europe and the United States, the assessment of coronary microvascular dysfunction (CMD) is recommended prior to an evaluation for coronary spasms [9]. In this procedure, NTG is administered before CMD evaluation, which means that a coronary spasm is induced after NTG. Although the induction rate of a coronary spasm after NTG is expectedly lower, such techniques are undoubtedly attracting attention.

The frequency of the unavoidable use of NTG among patients with VSA, as well as their clinical characteristics and prognoses, has not been clarified. Therefore, this study evaluated the clinical characteristics and prognosis of patients with VSA with a positive SPT who underwent the unavoidable use of NTG during the SPT.

## 2. Materials and Methods

### 2.1. Study Population

**Figure 1:**
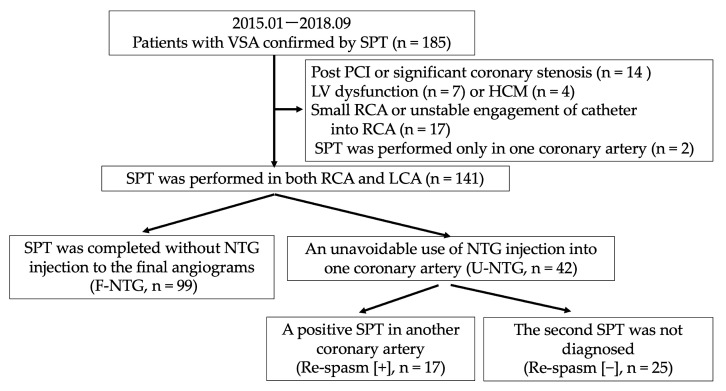
Flowchart of the study protocol. F-NTG, final use of nitroglycerin; HCM, hypertrophic cardiomyopathy; LCA, left coronary artery; LV, left ventricle; NTG, nitroglycerin; PCI, percutaneous coronary intervention; RCA, right coronary artery; SPT, spasm provocation test; U-NTG, unavoidable use of nitroglycerin; VSA, vasospastic angina.

This study was observational and retrospective. From January 2015 to September 2018, 233 patients were subjected to SPT at our institution to investigate chest pain at rest, during exertion, or in both settings. Of these patients, 185 (79%) were diagnosed with VSA by a positive SPT. We excluded patients a previous history of percutaneous coronary intervention or significant coronary stenosis (% stenosis > 50%; n = 14) or left ventricular wall motion abnormality (n = 7) or hypertrophic cardiomyopathy (n = 4). Until April 2016, spasm provocation was performed first in the RCA at our institution. Thereafter, the protocol was revised to start provocation with the LCA. To assess the presence of MVS, we also excluded patients in whom spasm provocation could not be performed in the RCA because of its small size or the inability to place a catheter into its ostium (n = 17). We also excluded two patients in whom only one of the coronary arteries was subjected to the SPT at the discretion of the attending physician (n = 2). In all, 141 patients were enrolled in the present study (Figure 1), of whom 99 completed SPT without receiving NTG for the final angiograms (F-NTG group). The remaining 42 (30%) received the unavoidable use of NTG with 0.3 mg intracoronarily due to prolonged coronary spasm or unstable haemodynamics due to coronary spasm and underwent a further SPT in another coronary artery after the unavoidable use of NTG administration (U-NTG group). All 141 patients underwent SPT performed in both the RCA and LCA. The study protocol was approved by the ethics committee at our institution. Written informed consent was obtained from all the patients.

### 2.2. SPT

We conducted the SPT using the previously published procedures [11,12,13,14]. Briefly, in the SPT for the RCA, 20 and 50 µg ACh were injected into the RCA following the initial coronary angiogram (CAG). A maximum dose of 80 µg ACh was injected into the RCA when a coronary spasm was not elicited by 50 µg ACh. In the SPT for LCA, 50 and 100 µg ACh were injected into the LCA. A maximum of 200 µg ACh was injected into the LCA if a coronary spasm was not induced by 100 µg ACh. For patients with probable active coronary spasms, a minimum dose of 20 µg was administered in the LCA. CAG was conducted shortly after a coronary spasm had been induced or after the maximum ACh infusion had been completed. Chest symptoms and electrocardiographic changes were evaluated at each induction. An SPT of the other coronary artery was performed without injecting NTG into the first coronary artery if a coronary spasm was elicited but resolved spontaneously. The unavoidable use of NTG was defined as an intracoronary injection of 0.3 mg NTG administered to reduce coronary spasms induced by ACh infusion into the first coronary artery if the spasms were persistent or severe enough to cause haemodynamic instability. The enrolled patients underwent a further SPT in another coronary artery after anunavoidable use of NTG. If a coronary spasm was not induced by the maximum ACh doses after the last CAG in the SPT for the other coronary artery, an intracoronary infusion of 0.3 mg of NTG was injected (F-NTG). Small dosages of catecholamines can be administered intracoronarily or intravenously if the patient’s haemodynamics remain unstable [15,16]. The low, moderate and high doses of ACh administered into the RCA and LCA were defined in this study as 20 and 50 µg, 50 and 100 µg, 80 and 200 µg, respectively.

### 2.3. Definition of Spasm-Related Factors

Coronary artery diameters were measured using the methodology described previously [17]. In brief, we selected spastic, atherosclerotic, and myocardial bridging (MB) segments for quantitative analysis. A single investigator who was blinded to the clinical data measured the luminal diameters in all cases using an end-diastolic frame in a computer-assisted coronary angiographic analysis system (CAAS II/QUANTCOR; Siemens, Berlin, Germany). Measurements were performed three times, and the average value was used for the analysis. Lesions with a stenosis of > 20% were classified as atherosclerotic lesions. Because MB has been associated with VSA [14,18], we examined MB, which was defined as a systolic constriction of the coronary artery diameter > 20% of the diastolic constriction. Variant angina (VA) was defined as angina with a spontaneous ST elevation on electrocardiogram (ECG). VSA was defined as a > 90% constriction of the coronary arteries on angiograms during the SPT, as well as the presence of characteristic chest pain and/or an ST-segment deviation on the ECG [3]. MVS was defined as coronary spasms that occur in two major coronary arteries, whereas SVS was defined as coronary spasms that occur in just one coronary artery. When the SPT for another coronary artery was negative after the unavoidable use of NTG, we could not diagnose the presence of MVS and the result was subsequently defined as ‘not diagnosed (ND)’. A focal spasm is defined as a temporary vessel narrowing > 90% within the boundaries of a single isolated coronary segment, as defined by the American Heart Association [7]. A diffuse spasm was defined as a > 90% diffuse vasoconstriction in two neighbouring coronary segments. The frequency of coronary spasms in the RCA left anterior descending coronary artery (LAD) and left circumflex coronary artery (LCX); frequency of low-dose acetylcholine (l-ACh)–induced spasms; total occlusion of a coronary artery (TOC) due to spasm; ST elevation on ECG; and severe complications, such as prolonged unstable haemodynamics requiring intravenous catecholamines, ventricular fibrillation and pulseless ventricular tachycardia, were checked.

### 2.4. Parameters

Data collected on the patients’ characteristics included current smoking status and any family history of coronary artery disease in their families. A systolic blood pressure > 140 mmHg, a diastolic blood pressure < 90 mmHg or taking an antihypertensive drug were all considered to be signs of hypertension. Prior to CAG, blood chemistry parameters were examined in the morning fasting condition. We evaluated total-cholesterol, triglycerides, high-density lipoprotein (HDL) cholesterol, low-density lipoprotein cholesterol, fasting blood sugar (FBS), haemoglobin A1c (HgA1c), C-reactive protein and brain natriuretic peptide levels. The existence of CKD was determined using accepted criteria [19] and the estimated glomerular filtration rate (eGFR, mL/min/1.73 m^2^) was computed using the standard formula [20]. Low-density lipoprotein cholesterol levels below 120 mg/dL or the usage of dyslipidaemia-related drugs were both deemed to indicate dyslipidaemia. An FBS of 126 mg/dL, HgA1c level of 6.5% or the usage of anti-diabetic drugs were considered as indicators of diabetes mellitus. The left ventricular ejection fraction (LVEF) was calculated using cardiac ultrasonography. The endothelium-dependent function of flow-mediated dilation (FMD) and the endothelium-independent function of NTG-induced dilation (NID) were evaluated as previously reported [12,21].

The parameters associated with VSA were examined, including VA frequency and the presence of other serious symptoms (such as cold sweating and syncope) or anginal attacks that occurred at rest, during exertion, or in both settings. Even though only one angina attack occurred in some cases, the number of angina attacks was still calculated based on the average number of angina attacks per month that may have occurred between the commencement of chest pain and hospital admission.

All study participants made at least one follow-up visit at our facility and patients were followed up as closely as was practical after discharge. The last data collection was performed in September 2022. Information from the medication diaries of patients who had recently made a follow-up visit was included in the follow-up assessments. We recorded the monthly number of coronary vasodilators used and angina events over the past 3 months. These evaluations were undergone by patients who could be evaluated at least 6 months after discharge (n = 111). The number of coronary vasodilators used was also assessed during hospital admission, discharge and final follow-up. For each patient, cardiac events, including readmission for angina or other cardiovascular problems, were recorded. Readmission or death from cardiac causes are deemed to be major adverse cardiac events (MACEs).

### 2.5. Statistical Analyses

Normally distributed data were expressed as mean ± standard deviation, whereas non-normally distributed data and non-continuous variables were expressed as median (interquartile range). The baseline characteristics of the groups were compared using Student’s unpaired t-test, Wilcoxon signed-rank test, or both. Logistic regression analysis was used to determine the unavoidable use of NTG. MACEs were analysed using the Kaplan–Meier survival curve and the log-rank test. JMP Ver. 16 (SAS Institute Inc., Cary, NC, USA) was used to perform all statistical analyses. Statistical significance was defined as *p* < 0.05.

## 3. Results

### 3.1. Patients’ Characteristics and VSA-Related Symptoms

The study included 42 patients with the unavoidable use of NTG (30%) and 99 patients without the unavoidable use of NTG (70%). The patients’ characteristics are presented in Table 1. The number of current smokers was significantly higher in the U-NTG group than in F-NTG group (*p* = 0.02). The frequencies of dyslipidaemia and taking statins did not vary significantly between the U-NTG and F-NTG groups. Regarding the blood chemical parameters, the level of high-density lipoprotein cholesterol (HDL-C) was significantly lower in the U-NTG group than in the F-NTG group (*p* = 0.02), whereas the other lipid parameters were comparable between the two groups. Regarding echographic parameters, no differences were found in LVEF, FMD and NID between the two groups (Table 2).

Regarding VSA-related symptoms, no significant differences were noted between the two groups in terms of chest symptoms that occurred at rest, during exertion, or in both settings and frequencies of VA and other serious symptoms, such as cold sweating and syncope. The number of anginal attacks per month was higher in the U-NTG group than in the F-NTG group (*p* = 0.04). The number of coronary vasodilators used during admission was similar between the two groups (Table 3). Logistic regression analysis for the presence of the unavoidable use of NTG using the current smoker status (odds ratio 3.3, *p* = 0.07), HDL-C concentration (odds ratio 2.9, *p* = 0.09) and the number of anginal attacks (odds ratio 1.8, *p* = 0.18) could not show the significant factor responsible for the unavoidable use of NTG.

### 3.2. CAG and SPT Findings

On the CAG, the frequencies of atherosclerotic lesions and myocardial bridge did not vary between the two groups (Table 4). For the SPT, no difference was observed between the two groups as to whether the SPT was initiated from the RCA or LCA. The frequency of MVS in the U-NTG group was 64%; other spasm provocation inductions were ND. The frequency of MVS in the F-NTG group was 55%, whereas that of SVS was 45%. The frequency of MVS did not differ between the two groups (*p* = 0.28). As for the coronary arteries in which spasm was provoked, no significant difference was found between the two groups for LAD (*p* = 0.12), but the frequencies of RCA and LCX (both *p* < 0.01) were greater in the U-NTG group than in the F-NTG group. No difference was found in the frequency of focal spasm between the two groups, but l-ACh-induced coronary spasms and ST-segment elevation during the SPT were more frequent in the U-NTG group than in the F-NTG group. Furthermore, the frequency of TOC due to coronary spasm tended to be higher in the U-NTG group than in the F-NTG group (*p* = 0.08). No differences in severe complications were found between the two groups (*p* = 0.83).

### 3.3. Prognosis

**Figure 2:**
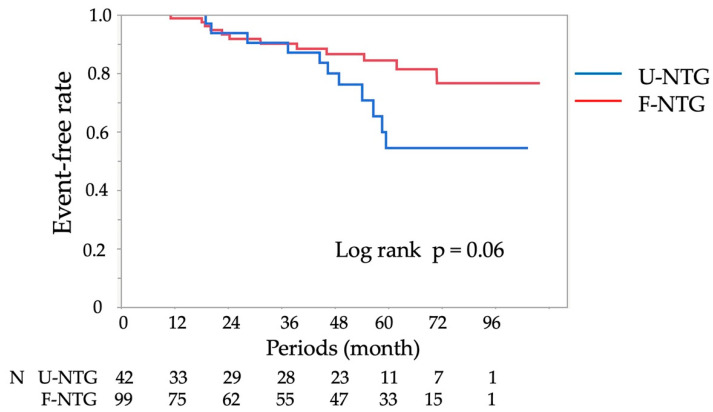
Kaplan–Meier curve for MACE-free survival during the follow-up period for the U-NTG and F-NTG groups. F-NTG, final use of nitroglycerin; MACE, major adverse cardiac event; NTG, nitroglycerin; U-NTG, unavoidable use of nitroglycerin.

The median follow-up period was 53 (20, 70) months. The average number of anginal attacks per month and the average number of coronary vasodilators at the final follow-up did not differ between the two groups (n = 31 and 80 in the U-NTG and F-NTG groups, respectively; Table 3). Confirmed MACEs included 1 case of cardiac arrest, 2 cases of heart failure, 3 cases of percutaneous coronary intervention, 16 cases of rehospitalisation due to unstable angina, 1 case of mitral valve plasty and 1 case of cerebrovascular disease. No difference was noted in the frequency of MACEs between the two groups, but a trend towards a lower frequency in U-NTG group was observed (log-rank, *p* = 0.06; Figure 2).

### 3.4. A Second SPT in Another Coronary Artery after an Unavoidable Use of NTG

**Figure 3:**
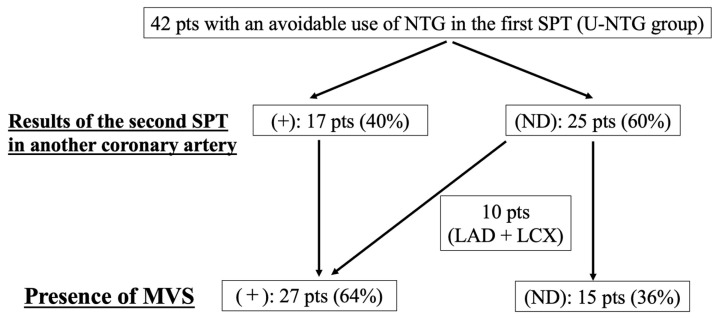
Flowchart of the SPT protocol for the 42 patients who underwent an unavoidable use of NTG in the first SPT. LAD, left anterior descending coronary artery; LCX, left circumflex coronary artery; ND, not diagnosed; NTG, nitroglycerin; SPT, spasm provocation test; U-NTG, unavoidable use of nitroglycerin.

In the U-NTG groups, the median time from an unavoidable use of NTG to the next SPT in another coronary artery was 8 ± 5 min. Among the 42 patients in the U-NTG group, 17 (40%) showed a positive SPT in another coronary artery (Re-spasm [+] group), whereas 25 showed a negative SPT (ND) in another coronary artery (Re-spasm [−] group), as shown in Figure 1 and Table 5. As a result, the frequency of MVS was 100% in the Re-spasm (+) group. Conversely, the frequency of MVS was 40% in the Re-spasm (−) group because this group included 10 patients who had already been diagnosed with MVS due to simultaneous coronary spasms in the LAD and LCX during the first SPT for LCA (Figure 3). In the Re-spasm (+) group, the patients tended to be older included (*p* = 0.05) and the frequency of current smokers was significantly lower (*p* < 0.01). NID on brachial sonography was significantly lower in the Re-spasm (+) group (*p* = 0.03), whereas the baseline brachial diameter and FMD were similar between the two groups. No differences were observed between the two groups in terms of which vessel was first provoked for SPT (*p* = 0.21) or the time between NTG administration and the next SPT (*p* = 0.33). The final dose of ACh did not differ between the two groups (*p* = 0.17). Logistic regression analysis for the presence of Re-spasm (+) using the current smoker status (odds ratio 2.7, *p* = 0.10) and NID (odds ratio 2.0, *p* = 0.16) could not show the significant factors responsible for the presence of Re-spasm (+).

## 4. Discussion

The present study evaluated the unavoidable use of NTG during SPT, including the frequency of the unavoidable use of NTG, as well as the clinical characteristics and prognoses of patients who underwent the unavoidable use of NTG. We found that the frequency of the unavoidable use of NTG was 30% in patients with positive SPT. Being a current smoker and having a low concentration of HDL-C may lead to the unavoidable use of NTG; however, the logistic regression analysis could not show statistical significance. We also observed higher vasospastic activity, including a higher number of chest symptoms on admission, higher frequencies of l-ACh–induced coronary spasms and ST elevation on ECG, in patients who underwent the unavoidable use of NTG. Nonetheless, the presence of MVS was diagnosed in 64% of patients in the U-NTG group without noting an increase in severe complications. Finally, the results of second SPTs in another coronary artery showed that smokers were less likely to test positive on the second SPT, even though they underwent the unavoidable use of NTG more frequently during the first SPT. ACh provocation test after NTG administration is currently receiving a lot of attention [9,10]. The findings of the present study may provide some guidance for future investigations.

The unavoidable use of NTG is an important intervention to avoid severe complications, such as ventricular fibrillation, during the SPT [3]. Our results showed that the unavoidable use of NTG was common, accounting for 30% of all cases. Sueda et al. [22] reported that the frequencies of the unavoidable use of NTG were 2.0% and 6.8% in the SPT using ACh and ergonovine maleate, respectively. The high frequency of the unavoidable use of NTG in the present study was due to the administration of NTG at our institution at the discretion of the chief operator. Although the higher frequency of ST elevation on ECG and a higher tendency of TOC due to coronary spasms may be a little upsetting to the operator, we always try to administer NTG immediately to be on the safe side. Thus, the frequency of the unavoidable use of NTG in the present study was clearly higher and may not be universally helpful. Nevertheless, this study showed that smoking and low HDL-cholesterolaemia may be possible risk factors for the unavoidable use of NTG, not to mention the risk of coronary spasm from smoking [5,6], which is listed as the first condition to be addressed [3] and is no doubt involved in the highly active state of coronary spasm. Conversely, some studies have shown that low HDL-cholesterolaemia is a risk factor for coronary spasm [23,24]; nevertheless, smoking has been found to cause low HDL-cholesterolaemia [25] and is likely to be the main risk factor in this study. In any case, further careful evaluation of risk factors for the unavoidable use of NTG needs to be conducted in future studies.

Our results also showed that patients who underwent the unavoidable use of NTG had an increased number of chest symptoms on admission and higher frequencies of l-ACh–induced coronary spasm and ST elevation on ECG during the SPT, indicating undoubtedly that such patients are more likely to have active coronary spasms. Regarding the prognosis of patients in the U-NTG group, the number of chest symptoms and those receiving coronary vasodilators were similar to those in the F-NTG group. However, the Kaplan–Meier curve for MACEs showed the relative difference at 4 years after discharge. These findings may mean that patients who underwent the unavoidable use of NTG should receive adequate doses of coronary vasodilators and be followed closely over the long term because of the high activity of coronary spasms.

ACh provocation after an intracoronary administration of NTG has garnered much interest [9,10]. Sueda et al. [22] reported that when other coronary arteries were provoked after NTG administration, 21.1% of patients were positive for ACh, whereas 52.9% were positive for ergonovine maleate. Several factors, such as the provocative agent is used, NTG dose, time interval from NTG administration, patient background and coronary artery re-provoked, may affect the positivity rate in the second SPT. In the present study, the positivity rate in the second SPT was 40% at 0.3 mg NTG with a mean interval of 8 min after the unavoidable use of NTG. The second SPT in our study had an average time interval of 8 min and no difference was found in the time interval between positive and negative cases in the second SPT. In addition, in the present study, smokers were fewer and NID was lower in the positive cases for the second SPT. Although the present study enrolled a small number of patients and we cannot fully explain the results, we hypothesise that smokers have a better response to NTG administration and are less likely to be positive on repeat SPT despite the high activity of coronary spasm that compels the unavoidable use of NTG in the first SPT. Brachial echocardiography is performed the day before SPT at our hospital and the response to NTG (NID) and smoking status may help predict positivity for the second SPT. Further studies involving a larger dataset are required. Sueda et al. stated that simultaneous positive results for LAD and LCX in the LCA indicates a diagnosis of MVS and SPT should be performed with the LCA for MVS evaluation [22]. In fact, in 10 of the 25 cases in which the second SPT was negative and ND for MVS, we were able to diagnose MVS because the first LCA provocation was simultaneously positive for LAD and LCX. We recognise the importance of starting SPT from the LCA first; however, since the LAD is the most likely to be positive among the coronary arteries, we found no difference in the positivity rate when the SPT was started from the RCA and then with the LCA in the second SPT. Our results suggest that initially provocatingthe RCA in patients with symptoms such as syncope may be acceptable, but the validity of this finding needs to be examined in more cases. Nonetheless, performing a second SPT for another coronary artery is important, even after an unavoidable use of NTG.

This study has several limitations. First, this study had a small sample size and was conducted retrospectively at a single institution. Particularly, the number of cases in which the second SPT was performed was small, which warrants a careful interpretation of the results. Second, the criteria for performing the unavoidable use of NTG and the time interval between the unavoidable use of NTG and second SPT was at the operator’s discretion and no precise protocol was established. To gain insights on SPT after NTG administration, it is necessary to determine a general protocol, including criteria for NTG administration and time to re-SPT and to perform re-SPT according to this protocol. Third, the follow-up rate was inadequate, which might have affected the prognoses. Furthermore, only the number of coronary dilators, type of drug [26], whether the drug was generic or not [27], and frequency of mediation were evaluated, which might have affected the chest symptoms. Fourth, among the 25 patients who were not induced by the second SPT, the final dose of ACh provocation was not high but rather moderate. The results might have been different if a higher dose had been used in these cases. Fifth, in some of the patients who underwent repeat SPT in the present study, those who already had coronary spasms in two vessels (LAD and LCX) and a diagnosis of MVS were evaluated to determine whether a coronary spasm would develop in the third coronary artery. However, the difference in prognosis between two-vessel and three-vessel coronary spasms among patients with MVS is not clear [13], and future studies are required to determine whether patients with two-vessel spasms should be evaluated for three-vessel spasms. Sixth, the methods in the present study include vascular response testing by brachial ultrasonography, which is not common. Therefore, the results of the present study may not imply that it is useful for the evaluation of all patients with VSA. Finally, chest symptoms are currently often assessed in a standardised and uniform manner using the Seattle Angina Questionnaire and other methods [28]. The average number of anginal attacks per month does not appear to be an accurate assessment of chest symptoms. A standardised method should be used in the future.

## 5. Conclusions

We evaluated the frequency of the unavoidable use of NTG during SPT and the characteristics of patients who received the intervention. We found that the unavoidable use of NTG was used in 30% of cases and that patients who received the unavoidable use of NTG were more likely to have active coronary spasms. We recommend that the unavoidable use of NTG should be used without hesitation to safely perform SPT. Furthermore, when the unavoidable use of NTG is used, a second SPT should be performed to evaluate for MVS. Although not definitively assessed in this study, smoking may be a predictor of the unavoidable use of NTG. However, once the unavoidable use of NTG is administered, the response is good and a second SPT may not be positive. Further studies involving a larger number of cases are needed to verify the study findings.

## Figures and Tables

**Table 1:** Patients’ characteristics.

	F-NTG	U-NTG	*p* Value
	99 (70)	42 (30)	
Male/female	40/59	23/19	0.12
Age (years)	67 ± 13	64 ± 12	0.20
Body mass index	23.8 ± 4.0	24.7 ± 4.6	0.21
Smoking	12 (12)	12 (29)	0.02
Hypertension	61 (61)	31 (74)	0.16
Dyslipidaemia	56 (57)	25 (64)	0.64
Diabetes mellitus	15 (15)	9 (21)	0.36
FH-CAD	14 (14)	8 (19)	0.46
CKD	27 (27)	13 (31)	0.66
Taking statins	45 (45)	19 (45)	0.98
Taking antiplatelet therapy	24 (24)	11 (26)	0.81

Numbers are expressed as number (percentage) or mean ± standard deviation. CKD, chronic kidney disease; F-NTG, final use of nitroglycerin; FH-CAD, family history of coronary artery disease; NTG: nitroglycerin; U-NTG, unavoidable use of nitroglycerin.

**Table 2:** Blood chemical and echographic parameters.

	F-NTG	U-NTG	*p* Value
Blood chemical parameters			
Total cholesterol (mg/dL)	202 ± 30	192 ± 41	0.13
Triglyceride (mg/dL)	141 ± 107	141 ± 84	0.98
HDL-cholesterol (mg/dL)	62 ± 8	55 ± 15	0.02
LDL-cholesterol (mg/dL)	107 ± 23	108 ± 31	0.77
Fasting blood sugar (mg/dL)	107 ± 23	108 ± 31	0.83
Haemoglobin A1c (%)	6.0 ± 0.6	6.0 ± 0.8	0.68
C-reactive protein (mg/dL)	0.06 (0.03, 0.16)	0.06 (0.03, 0.24)	0.96
eGFR (mL/min/1.73 m^2^)	69 ± 16	71 ± 16	0.51
Brain natriuretic peptide (pg/mL)	21 (12, 40)	20 (8, 33)	0.33
Echographic parameters			
LVEF on echocardiography (%)	68 ± 7	67 ± 7	0.41
FMD on brachial ultrasonography (%)	3.8 ± 2.4	4.0 ± 1.6	0.67
NID on brachial ultrasonography (%)	13.7 ± 6.9	15.1 ± 7.2	0.29

Numbers are expressed as number (percentage), mean ± standard deviation or median (interquartile range). eGFR, estimated glomerular filtration ratio; FMD, flow-mediated dilation; F-NTG, final use of nitroglycerin; HDL, high-density lipoprotein; LDL, low-density lipoprotein; LVEF, left ventricular ejection fraction; NID, nitroglycerin-induced dilation; NTG, nitroglycerin; U-NTG, unavoidable use of nitroglycerin.

**Table 3:** VSA-related parameters.

	F-NTG	U-NTG	*p* Value
Chest symptoms			
At rest/during exertion/both	77/11/11	37/2/3	0.34
VA (%)	0 (0)	1 (2)	0.12
Other serious symptoms (%)	18 (18)	10 (24)	0.44
Number of chest symptoms per month			
On admission	4 (2, 12)	9 (3, 15)	0.04
Follow-up	0 (0, 4) (n = 80)	0 (0, 1) (n = 31)	0.08
Number of taking vasodilators			
On admission	1 (0, 1)	0 (0, 1)	0.51
At discharge	1 (1, 1)	1 (1, 1)	0.26
Follow-up	1 (1, 2) (n = 80)	1 (1, 2) (n = 31)	0.49

Numbers are expressed as number (percentage) or median (interquartile range). F-NTG, final use of nitroglycerin; NTG, nitroglycerin; U-NTG, unavoidable use of nitroglycerin; VA, variant angina.

**Table 4:** The results of CAG and SPT.

	F-NTG	U-NTG	*p* Value
CAG			
Atherosclerotic lesion (%)	41 (41)	21 (50)	0.35
Myocardial bridging (%)	14 (14)	5 (12)	0.72
SPT			
Starting SPT from RCA/LCA	48/51	16/26	0.26
MVS (%)	54 (55)	27 (64)	0.28
Location			
RCA spasm (%, n/total n)	52 (51/99)	96 (24/25)	<0.01
LAD spasm (%, n/total n)	92 (92/99)	100 (35/35)	0.12
LCX spasm (%, n/total n)	23 (23/99)	52 (14/27)	<0.01
l-ACh-induced coronary spasm (%)	20 (20)	16 (38)	0.03
TOC due to coronary spasm (%)	4 (4)	5 (12)	0.08
Focal spasms (%)	47 (47)	20 (48)	0.99
ST elevation on electrocardiogram (%)	12 (12)	15 (36)	<0.01
Severe complications (%)	3 (3)	1 (2)	0.83
Vf/pVT (%)	3 (3)	0 (0)	0.25
Haemodynamic instability requiring catecholamines (%)	0 (0)	1 (2)	0.12

Numbers are expressed as number (percentage). CAG, coronary angiography; F-NTG, final use of nitroglycerin, l-ACh, low-dose acetylcholine; LAD, left anterior descending coronary artery; LCA, left coronary artery; LCX, left circumflex coronary artery; MVS, multi-vessels spasm; NTG, nitroglycerin; pVT, pulseless ventricular tachycardia; RCA, right coronary artery; SPT, spasm provocation test; TOC, total occlusion of a coronary artery; U-NTG, unavoidable use of nitroglycerin; Vf, ventricular fibrillation.

**Table 5:** Results of a second SPT in another coronary artery.

	Re-Spasm (−)	Re-Spasm (+)	*p* Value
Number (%)	25 (60)	17 (40)	
Male/Female	16/9	7/10	0.14
Age (years)	61 ± 11	68 ± 12	0.05
Current smoker (%)	11 (44)	1 (6)	<0.01
Frequency of coronary vasodilators (%)	8 (32)	8 (47)	0.33
Brachial ultrasonography			
Baseline brachial diameter (mm)	4.0 ± 0.7	4.1 ± 0.7	0.81
FMD (%)	3.9 ± 1.6	4.1 ± 1.6	0.68
NID (%)	16.9 ± 7.2	11.2 ± 5.9	0.03
SPT			
Starting SPT from RCA/LCA	7/18	8/9	0.21
Time from an unavoidable use of NTG to the second SPT (min)	7 ± 6	8 ± 4	0.33
MVS (%)	10 (40)	17 (100)	<0.01
Final doses of ACh in another coronary artery: low/moderate/high	0/15/10	1/13/3	0.17

Numbers are expressed as number (percentage) or mean ± standard deviation. ACh, acetylcholine; FMD, flow-mediated dilation; LCA, left coronary artery; MVS, multi-vessel spasm; NID, nitroglycerin-induced dilation; NTG, nitroglycerin; RCA, right coronary artery; SPT, spasm provocation test.

## Data Availability

Not applicable.
